# TcCYPR04, a Cacao Papain-Like Cysteine-Protease Detected in Senescent and Necrotic Tissues Interacts with a Cystatin TcCYS4

**DOI:** 10.1371/journal.pone.0144440

**Published:** 2015-12-07

**Authors:** Thyago Hermylly Santana Cardoso, Ana Camila Oliveira Freitas, Bruno Silva Andrade, Aurizangela Oliveira de Sousa, André da Silva Santiago, Daniela Martins Koop, Karina Peres Gramacho, Fátima Cerqueira Alvim, Fabienne Micheli, Carlos Priminho Pirovani

**Affiliations:** 1 Centro de Biotecnologia e Genética, Universidade Estadual de Santa Cruz, UESC, Rodovia Ilhéus-Itabuna, Km 16, Ilhéus/BA, Brasil; 2 Universidade Estadual do Sudoeste da Bahia, UESB, Jequié-BA, Brazil; 3 Centro de Pesquisa do Cacau (CEPEC), Comissão Executiva do Plano da Lavoura Cacaueira (CEPLAC), Fitopatologia, Ilhéus, BA, Brazil; 4 CIRAD, UMR AGAP, Montpellier, France; Russian Academy of Sciences, Institute for Biological Instrumentation, RUSSIAN FEDERATION

## Abstract

The interaction amongst papain-like cysteine-proteases (PLCP) and their substrates and inhibitors, such as cystatins, can be perceived as part of the molecular battlefield in plant-pathogen interaction. In cacao, four cystatins were identified and characterized by our group. We identified 448 proteases in cacao genome, whereof 134 were cysteine-proteases. We expressed in *Escherichia coli* a PLCP from cacao, named TcCYSPR04. Immunoblottings with anti-TcCYSPR04 exhibited protein increases during leaf development. Additional isoforms of TcCYSPR04 appeared in senescent leaves and cacao tissues infected by *Moniliophthora perniciosa* during the transition from the biotrophic to the saprophytic phase. TcCYSPR04 was induced in the apoplastic fluid of Catongo and TSH1188 cacao genotypes, susceptible and resistant to *M*. *perniciosa*, respectively, but greater intensity and additional isoforms were observed in TSH1188. The fungal protein MpNEP induced PLCP isoform expression in tobacco leaves, according to the cross reaction with anti-TcCYSPR04. Several protein isoforms were detected at 72 hours after treatment with MpNEP. We captured an active PLCP from cacao tissues, using a recombinant cacao cystatin immobilized in CNBr-Sepharose. Mass spectrometry showed that this protein corresponds to TcCYSPR04. A homology modeling was obtained for both proteins. In order to become active, TcCYSPR04 needs to lose its inhibitory domain. Molecular docking showed the physical-chemical complementarities of the interaction between the cacao enzyme and its inhibitor. We propose that TcCYSPR04 and its interactions with cacao cystatins are involved in the senescence and necrosis events related to witches’ broom symptoms. This molecular interaction may be the target for future interventions to control witches' broom disease.

## Introduction

Proteases (also known as peptidases, proteinases or proteolytic enzymes) are mainly divided in two groups: i) exoproteases, which cleave at the ends of the protein and are subdivided into amino- or carboxyproteases; and ii) endoproteases, cleaving on the interior of the protein. The classification of endoproteases depends on the type of residue at the active site and according to MEROPS database (http://merops.sanger.ac.uk/index.shtml) it includes: aspartic (Asp residue), cysteine (Cys), glutamic (Glu), metallo (metal ion), asparagine (Asn), serine (Ser), and threonine (Thr) proteases, and mixed (Cys, Ser, Thr) and unknown (unknown catalytic type) classes [1]. Cysteine-proteases consist of a single chain folded to form two domains—an α-helix-rich and a β-barrel-like domain—separated by a cleft containing the active site formed by the Cys and His residues, each one on each domain [2]. For proteolysis, this Cys acts as a nucleophile activated by His in the active site [3, 4]. To date, proteases are represented by 220 families belonging to 13 clans, whereof 72 families and 8 clans correspond to cysteine-proteases [1]. Papain-like cysteine-proteases (PLCPs) are grouped in clan CA, family C1. PLCPs are usually 23–30 kDa in size, and contain an inhibitory pro-region folded back onto the substrate-binding cleft; this inhibitory region needs to be removed for protease activation.

Many plant PLCPs are produced with a signal peptide indicating that they are secreted in the endomembrane system [4]. In plants, PLCPs are involved in pathogen perception, disease resistance, defense against insects and senescence [4]. Proteolysis during plant-pathogen interactions probably promotes the selection of counteracting inhibitors, non-cleavable substrates and other means to evade proteolysis [5, 6]. Therefore, the interaction of proteases with their substrates and inhibitors can be seen as a molecular battlefield [7]. The structure of the complex between exogenous papain and cystatin is well analyzed [8, 9], but little is known about the interaction between PLCP and cystatin in the same organism.

Cystatins are divided into four families: i) family-1 cystatins (stefin family) composed of proteins devoid of sulfur bridges. This group has a molecular mass of about 11 kDa and is generally present in the cytosol; ii) family-2 cystatins (cystatin family), comprising proteins that have sulfide bridges; iii) family-3 cystatins (kininogen family) characterized by high molecular-weight glycoproteins with three repetitions. These proteins contain tandem domains that result from the gene duplication of the family–2 cystatins, and finally; iv) family–4 cystatins (phytocystatins), which includes all cystatins described for plants to date [10, 11].

Phytocystatins are specific plant proteins that inhibit PLCPs; they are small, mostly ranging from 12 to 16 kDa. Several roles have been attributed to phytocystatins, since the regulation of various endogenous proteolytic processes to the inhibition of exogenous cysteine-proteases secreted by predatory or pathogenic organisms during herbivory or infection [12, 13]. Previous work performed by our group identified and characterized four phytocystatins [14] from *Theobroma cacao* L.-*M*. *perniciosa* interaction cDNA libraries [15]. These cystatins, named TcCys1 to TcCys4, were expressed in *E*. *coli* and the recombinant proteins displayed inhibitory activity *in vitro* against commercial papain, and antifungal activity against *M*. *perniciosa* [14]. TcCYS4 accumulates when green broom symptoms occur, probably inhibiting the cysteine-proteases involved in programmed cell death in infected tissues [16].

In this work we have produced a polyclonal antibody against a PLCP (TcCYSPR04—E.C. 3.4.22.16) identified in cDNA libraries from the interaction between *T*. *cacao* and *M*. *perniciosa*, and analyzed the accumulation of the protein in different cacao tissues by Western blot. The TcCYS04 accumulation was also investigated in tobacco leaves treated with recombinant ethylene-inducing protein from *M*. *perniciosa* (MpNEP) [17, 18]. Recombinant sepharose-immobilized cacao cystatin captured an active PLCP from cacao tissue, whose was confirmed by mass spectrometry as TcCYSPR04. The structures of TcCYSPR04 and TcCYS4 were obtained by homology modeling method. The interaction of proteins was predicted by computational docking. Our data indicate that the interaction between protease (TcCYSPR04) and cystatin (TcCYS4) are involved in the senescence and necrosis events that can be triggered in development of witches' broom symptoms; therefore, the balance between these proteins could be exploited for controlling the disease.

## Material and Methods

### Plant material and Protein extraction

Primordial leaves of Catongo cacao susceptible genotype (seedlings aged 20 days after plantation) were inoculated with a suspension containing 200.000 basidiospores x mL^-1^ of *M*. *perniciosa* [19] or water (control), and kept in a greenhouse until the end of the experiment. Leaves were harvested at three stages after inoculation: E1—leaves up to 10 days; E2—leaves for 15–25 days (without necrosis spots visible); E3—leaves with more than 35 days (necrosis spots visible). Leaves without visible injury (young leaves) and leaves with chlorotic appearance (senescent leaves), as well as mature leaves infected with *M*. *perniciosa* with absence of necrosis (green brooms) from Catongo variety were collected in an experimental field at UESC—Ilhéus, Bahia, Brazil. All the collected samples were used to total protein extraction [20].

At experimental field, we also collected leaves from healthy and infected Catongo (CAT) and TSH1188 (TSH), a resistant genotype, to perform the apoplastic fluid proteins extraction, according to Pirovani et al [20]. Tobacco (*Nicotiana tabacum*) plants were used to necrosis assay induced by NEP [17] in leaves. For this, tobacco leaves maintained under aseptic conditions were sprayed with 1.4 μM of recombinant NEP from *M*. *perniciosa* (MpNEP) [18] and collected at 24, 36, 40, 60 and 72 hours after spraying. As experimental control were used tobacco leaves maintained in the same conditions, but it not treated with MpNEP. Total protein extracts were obtained from these leaves [20].

### Quantitative evaluation and classification of proteases in the genome of cocoa

Proteases sequences were retrieved using the database of the genome sequence of a Belizian Criollo genotype (B97-61/B2) provided by the International Cocoa Genome Sequencing Consortium [21]. The identification of proteases was done by searching for “keywords” in the database containing the BLAST analysis results of total genomic sequences of *T*. *cacao* against *Arabidopsis thaliana*, *Populus trichocarpa*, *Glycine max* and *Vitis vinifera*. Initially, the words “protease”, or “proteinase” or “peptidase” have been used and then a screening was performed by analyzing each result in order to eliminate those corresponding to other proteins, but containing the keywords, as in the case of protease inhibitors and proteases targets.

### Sequence analysis

The fragment of cysteine-protease pre-pro-TcCYSPR04 (E.C. 3.4.22.16) and the cystatin TcCYS4 sequences were identified in expressed sequence tags (EST) libraries from the cacao-*M*. *perniciosa* interaction [15]. The complete sequence of TcCYSPR04 was identified in the EST data bank from Cirad/France (http://esttik.cirad.fr/) [21] and confirmed in CocoaGenDB (http://cocoagendb.cirad.fr/gbrowse/cgi-bin/gbrowse/theobroma/) [22]. The sequences were compared for putative function determination and annotation to the public sequence database (http://www.ncbi.nih.gov/BLAST/) using BLASTX and TBLASTX. Alignments showing similarity to an expected value lower than 1.10^−4^ were considered significant.

Protein translation was carried out using Translate tool (http://au.expasy.org/tools/dna.html); the open reading frame (ORF) was defined using the ORF Finder program (http://www.ncbi.nlm.nih.gov/gorf/gorf.html); peptide signal was predicted using SignalP (http://www.cbs.dtu.dk/services/SignalP/); and the functional domains of the proteins were identified using the Pfam software (http://www.ebi.ac.uk/Tools/InterProScan/) and the MEROPS database (http://merops.sanger.ac.uk/index.shtml) [23]. The ExPASy tools (http://www.expasy.org) were used to determine the molecular mass and pI of the proteins (http://www.expasy.org/cgi-bin/pi_tool), the putative phosphorylation sites (NetPhos 2.0, http://www.cbs.dtu.dk/services/NetPhos/) and the putative glycosylation sites (NetGlycate 1.0 Server and OGPET v1.0, http://www.geneinfinity.org/sp/sp_proteinptmodifs.html; YinOYang 1.2, http://www.cbs.dtu.dk/services/YinOYang/). Prediction of the secondary structures of proteins was obtained using the PSIPRED-GenThreader (http://bioinf.cs.ucl.ac.uk/index.php?id=780).

### Expression of recombinant cysteine-proteases and antibody production

The ORFs encoding cysteine-protease proteins were obtained by amplification, using the following forward and reverse primers: TcCYSPRot_CatF GTTTCAGAAACATATGTTGGGAGCTGC and TcCYSPRotR AACCTCAACCCTCGAGATGGACCAACTAC, which contained restriction sites for *Xho*I and *Nde*I, respectively, for cloning into pET28a, according to standard techniques for cloning [24]. Transformed cells (*E*. *coli* Rosetta—DE3) containing the recombinant plasmids were grown at 37°C until reaching OD_600nm_ = 0.7, induced with 0.4 mM IPTG (isopropyl-β-D-thiogalactopyranoside) for 4 h, harvested, and processed. The lysate was centrifuged at 13,000 g, 4°C, for 15 min and soluble and insoluble fractions were obtained. Fusion proteins with a histidine tail were purified using a His-Trap FF Crude column (GE Healthcare), following the manufacturer’s instructions. Insoluble recombinant cacao cysteine-proteases were dissolved with buffer solution 6 M urea prior to loading onto the column, and eluted in lyses buffer containing 250 mM imidazole and 6 M urea. Protein concentration was determined by the Bradford method [25].

### Antibody production

Anti-TcCYSPR04 polyclonal antibody was obtained by rabbit immunization with the purified recombinant protein (His-tagged TcCYSPR04), according to Pirovani et al [14]. The rabbit was maintained under standard conditions in the animal house at UESC. All immunizations were performed in accordance with protocols approved by the Ethics Commission in Animal Experimentation (CEUA—UESC) [Protocol number 025/09].

The antibody obtained was purified by affinity using the His-tagged TcCYSPR04 antigen immobilized in nitrocellulose membrane [24]. The specificity of the anti-TcCYSPR04 serum was previously evaluated with protein extracts to determine its titer, whereas immunoblotting experiments were conducted at a 1:4000 dilution of this serum.

### Immunodetection of cysteine-proteases in cacao and tobacco tissues

Total proteins were extracted under native conditions, as described by Pirovani et al [20]. For Western blot analyses, equal amounts (5 μg) of each protein samples were separated by 15% SDS-PAGE and electroblotted onto Hybond-C Extra membrane (Amersham Biosciences, Buckinghamshire, UK). The protein blot was blocked with 5% casein in TBS-T buffer (20 mM Tris–HCl pH 7.6; 0.8% NaCl; 0.1% Tween 20) and incubated with specific polyclonal anti-TcCYSPR04 antibody, for 1 h, at room temperature. An alkaline phosphatase-labeled anti-rabbit antibody (Life Technologies, Carlsbad, CA, USA) was used as secondary antibody (dilution 1:5,000) [24]. The detection system was NBT/BCIP (Promega, Madison, Wisconsin, USA).

### Protease trap

Capture system were used with BSA (control) and recombinant cystatins TcCYS3 and TcCYS4 previously cloned and expressed [14] coupled to CNBr-activated Sepharose TM 4 fast Flow (GE Healthcare), according to the manufacturer instructions. The protease capture was performed using total proteins extracted of the tissues cacao from Catongo variety as described by Pirovani et al [14]. The captured proteases were analyzed regarding protease activity by a qualitative assay. For this, 15% SDS-PAGE and 7% PAGE with ampholytes (IEF—pH 4.0–8.0) were used. After migration, the gels were washed in Triton X-100, at room temperature, for 30 min for SDS removal and protease renaturation. After then, the gels were overlaid with 8% polyacrylamide-0.1% gelatin-gel in the presence of protease activity buffer at room temperature, for 12h, as described above [26, 27]. Activity gels were stained with 0.01% Coomassie blue G-250 [28].

### Mass Spectrometry Identification of the captured protease

The captured proteases were mixed with 25 mM of NH_4_HCO_3_ and 10 mM of dithiothreitol (DTT), and kept at 60°C for 30 min. Then, 50 mM of iodoacetamide were added to the sample, which was kept at room temperature, for 30 min, in the dark, and finally digested with trypsin (25 ng/μl) at 37°C, for 12h, according to the manufacturer’s instructions (Promega). The resulting tryptic digests were vacuum concentrated (Concentrator 5301, Eppendorf), desalted using a pre-Symmetry column (Waters, Mildford, MA, USA) C18 (5 μm, 180 μm in inner diameter x 20 mm long), and then fractioned by C18 reverse phase chromatography column (100 mm x 100 μm, 1.7 μm particles) on the nanoAcquity UPLC (WATERS), for 50 min, under 0.6 μL.min^-1^ acetonitrile flux. The following gradient was used: 1% for 1 min, 1%-50% in 40 min, 50%-85% in 5 min, 85% for 2 min, 85%-1% in 1 min, 1% for 2 min. Afterwards, the peptides were deionized at 3,000 V and split on positive mode, with a minimum relative intensity of 10 counts on Micromass ESI-Q-TOF (WATERS). Spectra were analyzed using the ProteinLynx Global Server 4.2 (WATERS) and compared with the *T*. *cacao* CocoaGenDB genomic database (http://cocoagendb.cirad.fr/gbrowse/cgi-bin/gbrowse/theobroma/).

### 3D model building

The 3D models of TcCYSPR04 and TcCYS4 were built using a comparative modeling approach. The sequences were subjected to the BLASTP (http://www.ncbi.nlm.nih.gov/BLAST) [29] restricted to the Protein Data Bank (PDB, http://www.pdb.org/), and used the Blossum62 align matrix [30] for template identification. Alignments showing similarities higher than 44% and 38.42%, respectively, and an expected value lower than 1.10^−5^ were considered significant. The 3D models were constructed using the SwissPdb Viewer v.3.7 [31, 32], following the standard protocol: i) load the template PDB file; ii) align the primary target sequence with template; iii) submit modeling request to the Swiss Model Server [33].

### Structure refinement and molecular dynamics simulation

For structure refinement and molecular dynamics (MD) simulation, the LEAP [34] and SANDER [35] utilities of AMBER v.14 have been used [36]. The initial models were neutralized using LEAP, and submitted to SANDER for structure refinement. In addition, the model structures were fully minimized with 1000 steps of steepest descent minimization followed by 1000 more steps of conjugate gradient to an RMSD gradient of 0.01 kcal/2.71Å in vacuum, and then in water, for 1000 steps of steepest descent followed by 1000 more steps of conjugate gradient to an RMSD gradient of 0.01 kcal/2.71Å. After that, MD simulations of the refined structures were performed in water, using ff12SB force field at 300 K for 5 ns. All MD simulations were carried out without constrain methods. The cutoff value of 10 Å was used for minimization of geometry and MD simulations. All the MD steps were visualized on PyMol 1.7.4.4 and VMD 1.9.2 [37].

### Validation of the 3D structure

PROCHECK 3.4 [38] and ANOLEA (Atomic Non-Local Environment Assessment) [39] were used to evaluate both final models by evaluating their Ramachandran plots [40] and the energy plots from each heavy atom in the molecule, respectively [41].

### Docking

The docking between 3D models of TcCYSPR04 and TcCYS4 was made using the ClusPro 2.0 web Server (http://cluspro.bu.edu/) [42] and the FFT Docking [43] softwares, which are specific for protein-protein docking. Cluspro is the first fully automated web-based program for protein docking and is considered one of the best software by the international scientific community (e.g. CAPRI-Critical Assessment of Predicted Interactions) [44]. After estimating the energy (van der Walls, electrostatic and hydrophobic forces) and the size of the cluster—preferring lower energies and larger cluster sizes—we selected one of the returned models (among 85). The interactions were refined by analyzing the TcCYS4 residues exposition, the orientation of TcCYS4 in relation to TcCYSPR04, and the conformation energy profile. Finally, the best model of the TcCYSPR04-TcCYS4 complex was compared with the PDB file of the resolved structure of catepsin/stefin complex [45].

## Results

### Proteases in the genome of cocoa and sequence analysis of TcCYSPR04

In the 28,798 proteins encoded by the cacao genome [22], 448 proteases have been identified. These proteases were distributed in five classes recognized by the KEGG database (Kyoto Encyclopedia of Genes and Genomes) according to the amino acid present in the active site. For class metallo-, aspartic-, serine-, cysteine- and threonine-proteases, 67, 63, 157, 134 and 27 proteins have been identified, respectively.

**Fig 1:**
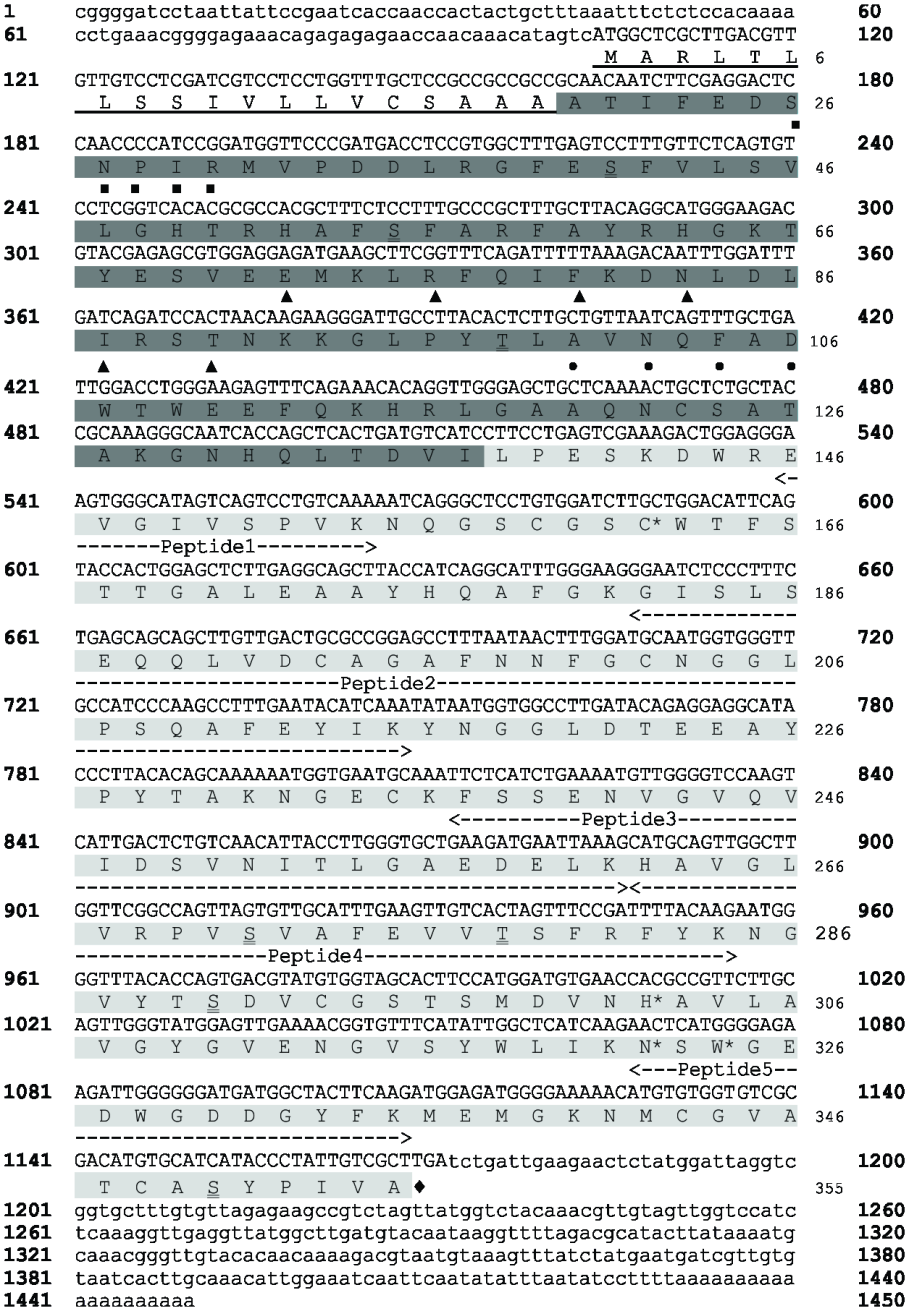
Nucleotide and amino acid sequences of pre-pro-TcCYSPR04. Nucleotides of ORF and non-coding regions are shown in uppercase and lowercase letters, respectively. Signal peptide is underlined. White letters on dark gray background indicate the pro-region of the protein. The light gray region represents the catalytic region of the protein. Putative phosphorylated amino acids are indicated in boxes. The double underline shows the putative glycosylated amino acids. ■ indicates the vacuolar targeting motif SNPIR. ▲ and • indicates the ERFNIN and ANFD motifs of the pro-region. Amino acids from the catalytic site are indicated by *. Peptides identified by mass spectrometry after capture by TcCYS4 immobilized on CNBr-sepharose resin are indicated. ♦ indicates the stop codon.

The TcCYSPR04 fragment was previously identified in a cacao-*M*. *perniciosa* interaction cDNA library [15]. The complete nucleotide and amino acid sequences identified [22] revealed an ORF of 1068 nucleotides encoding a protein of 355 amino acid residues (Fig 1). A predicted hydrophobic N-terminal region corresponding to the signal peptide in the protein sequence has been detected between the amino acids M1 and A19, and an inhibitory region (pro-region) was found to exist between the amino acids A20 and I137.

The pro-region contained the strongly conserved SNPIR, ERFNIN (EX_3_RX_3_FX_2_NX_3_IX_3_N) and ANFD (AXNXFXD) motifs. The catalytic region of the protein was comprised between the amino acids L138 and A355. The predicted molecular mass and pI of the different protein forms were: i) 39 kDa and 5.43 for the pre-pro-TcCYSPR04 (355 amino acids); ii) 37 kDa and 5.32 for the pro-TcCYSPR04 (336 amino acids); iii) 23.4 kDa and 4.63 for the TcCYSPR04 (218 amino acids).

Fourteen putative phosphorylation sites were detected: six on serine residues (S55, S69, S184, S238, S271 and S279), five on threonine residues (T21, T90, T108, T289, T296), and three on tyrosine residue (Y67, Y226 and Y333). Seven putative glycosylation sites were detected: five on serine (S41, S55, S271, S290 and S350) and two on threonine (T98 and T278).

### Immunodetection of cystein proteinases in cacao tissues

**Fig 2:**
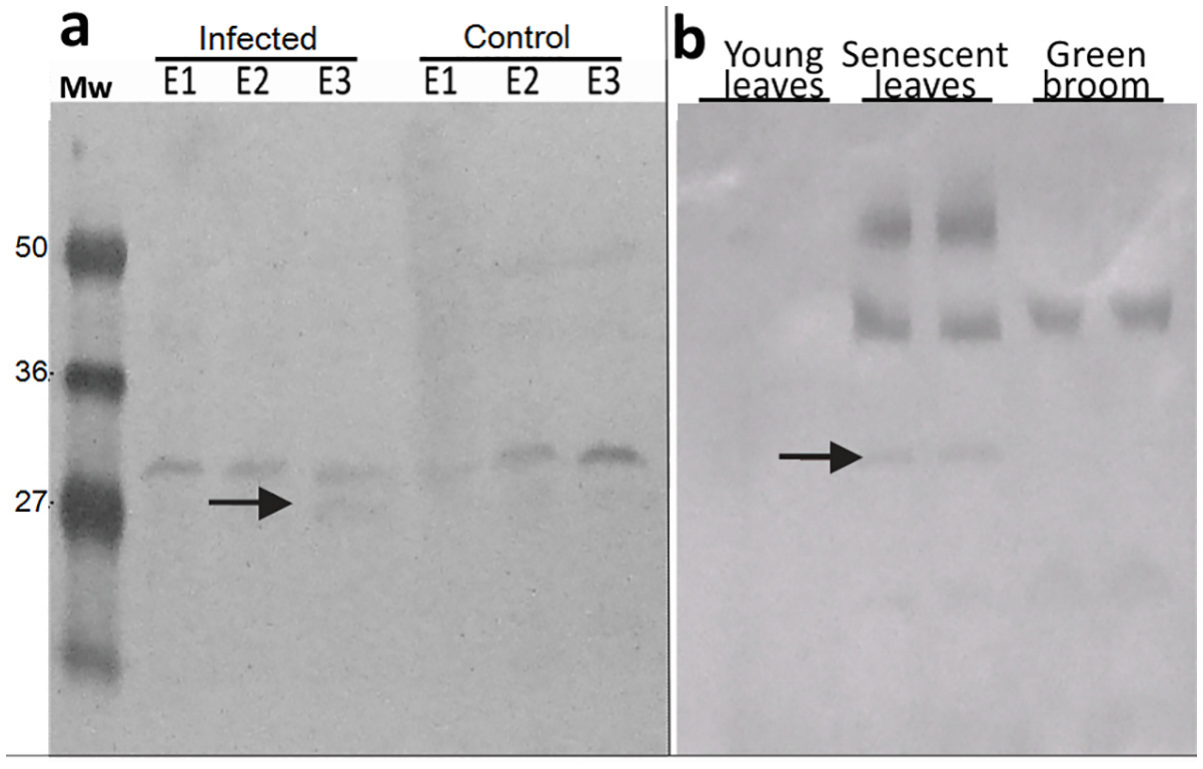
TcCYSPR04 in protein extracts from cacao. **a** Immunodetection of TcCYSPR04 at different plant development stages reported to Catongo cultivated in greenhouse. “Infected” denotes plants whose the leaf primordia were inoculated with basidiospores of the fungus *M*. *perniciosa*, whereas “control” stands for healthy plants. E1—leaves up to 10 days after inoculation (DAI); E2—leaves 15–25 DAI; E3—leaves with more than 35 DAI (early necrosis). The arrow indicates an additional protein band with molecular mass ~27 kDa, exclusively detected in E3 stage of the infected plants. **b** Immunodetection of TcCYSPR04 in extracts of cacao grown under field conditions. Young leaves—no visible lesions; senescent leaves—leaves with chlorotic appearance; green broom—mature leaves infected with *M*. *perniciosa* with absence of necrosis, collected in the field. The arrow indicates protein band with molecular mass ~30 kDa detected in senescent leaves from *T*. *cacao*. Mw—protein molecular weight marker in kDa.

TcCYSPR04 was immunodetected in cacao tissues of the susceptible variety (Catongo), control and inoculated with *M*. *perniciosa* on three stages after inoculation (E1, E2 e E3). A band with an expected size of ~30 kDa corresponding to the mature protein was observed in all stages analyzed for control and inoculated plants (Fig 2a). The intensity of the bands increased from E1 to E3 stages in control plants (healthy). Already for the infected plants, we observed an inverse situation for TcCYSPR04 accumulation. The band intensity decreased from E1 to E3 stages in infected plants; however, an additional band with ~27 kDa was detected in E3 (Fig 2a, arrow).

Immunodetection performed to protein extracts of plants from experimental field revealed bands in senescent leaves and green broom but not in young leaves, (Fig 2b). Senescent leaves showed the expected band with ~30 kDa, but other bands were observed also in this sample (Fig 2b, arrow). High intensity bands with molecular mass higher than 36 kDa and 50 kDa were detected in senescent leaves, whereas in green broom only the band higher than 36 kDa was observed (Fig 2b).

**Fig 3:**
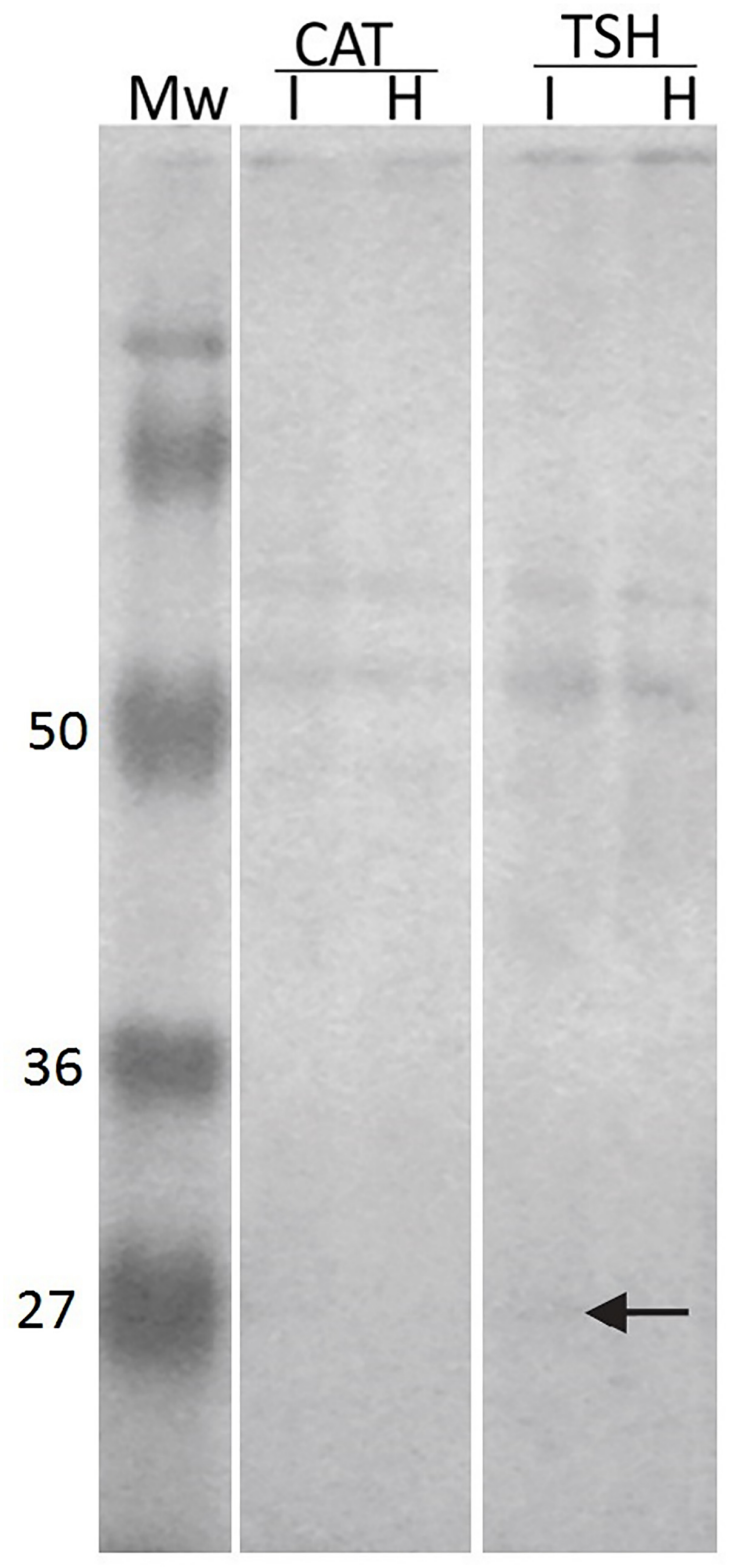
TcCYSPR04 in apoplastic fluid from cacao. Immunodetection of TcCYSPR04 in apoplastic fluid of leaves from susceptible and resistant varieties of cacao. CAT—cacao genotype Catongo susceptible to *M*. *perniciosa*. TSH—cacao genotype TSH1188 resistant to *M*. *perniciosa*. I—apoplastic fluid extracted from leaves infected with *M*. *perniciosa*. H—apoplastic fluid extracted from healthy leaves. Mw—protein molecular weight marker in kDa. The arrow indicates protein band with molecular mass ~27 kDa detected in infected apoplastic fluid from TSH1188.

In the apoplastic fluid, bands of about 50 kDa were detected in both conditions, healthy and infected, analyzed from susceptible (Catongo) and resistant (TSH1188) varieties (Fig 3). The protein bands were more intense in the apoplastic fluid extracted from infected leaves as compared to the healthy leaves. However, TSH1188 apoplastic fluids (infected and healthy) showed bands more intense than Catongo apoplastic fluids. In the infected apoplastic fluid from TSH1188, a band with ~27 kDa was found (Fig 3, arrow).

**Fig 4:**
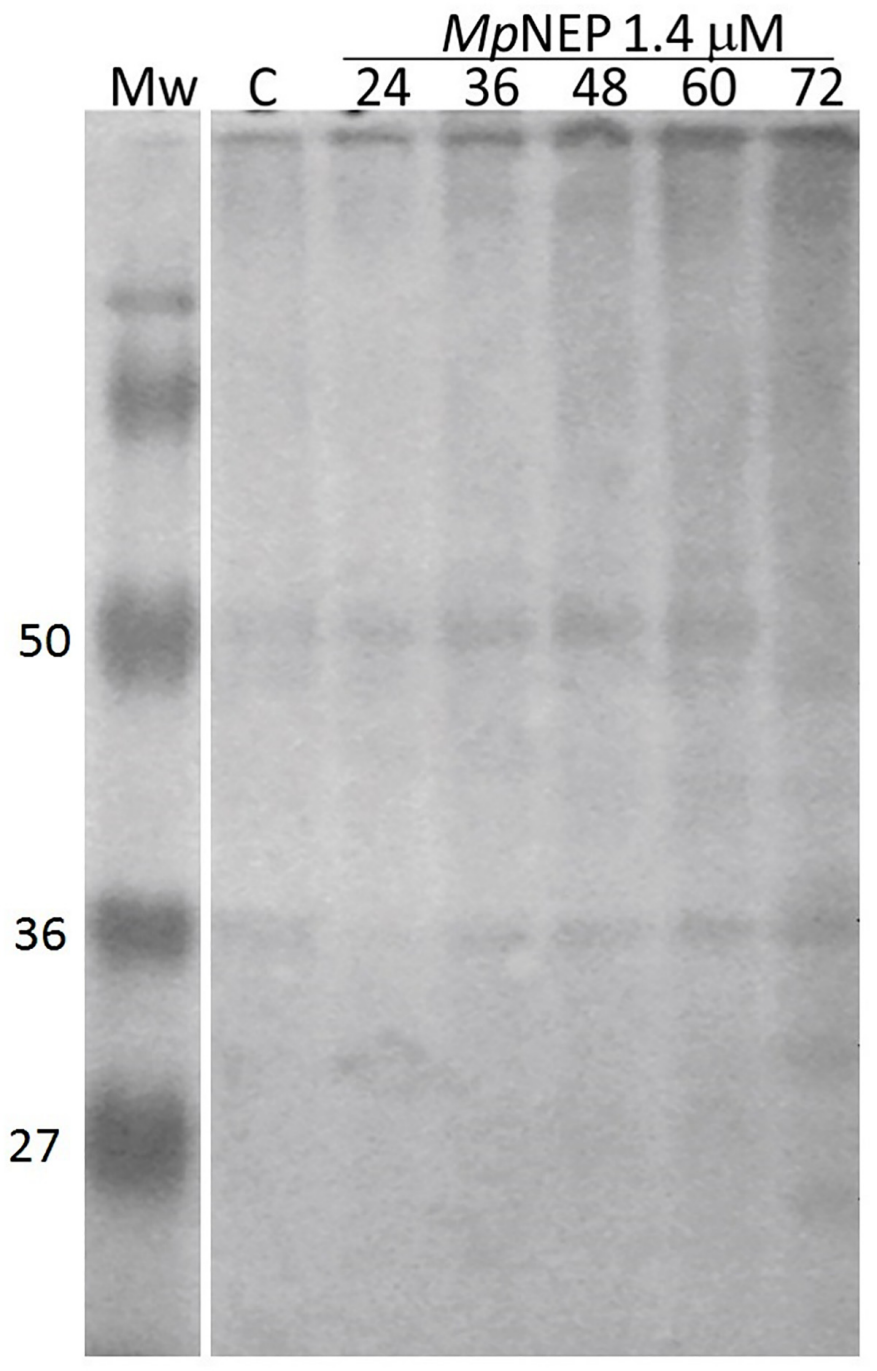
TcCYSPR04 in tobacco leaves treated with MpNEP. Immunodetection of TcCYSPR04 in protein extract from tobacco leaves treated with 1.4 μM of recombinant protein NEP from *M*. *perniciosa*. C—protein extract from untreated leaves. 24, 36, 40, 60 and 72—hours after spraying the leaves with MpNEP. Mw—protein molecular weight marker in kDa.

TcCYSPR04 was immunodetected in tobacco leaves treated with MpNEP. The cross-reaction showed bands with molecular mass between 36 and 50 kDa in all samples analyzed, inclusive in control sample (Fig 4). An exclusive band with the expected size for mature protease between 25–35 kDa was observed in sample extracted at 72 hours after treatment with MpNEP. At 72 hours after treatment with MpNEP, also was detected a band with molecular mass less than 27 kDa (Fig 4).

### Protease trap and activity analysis on gel

**Fig 5:**
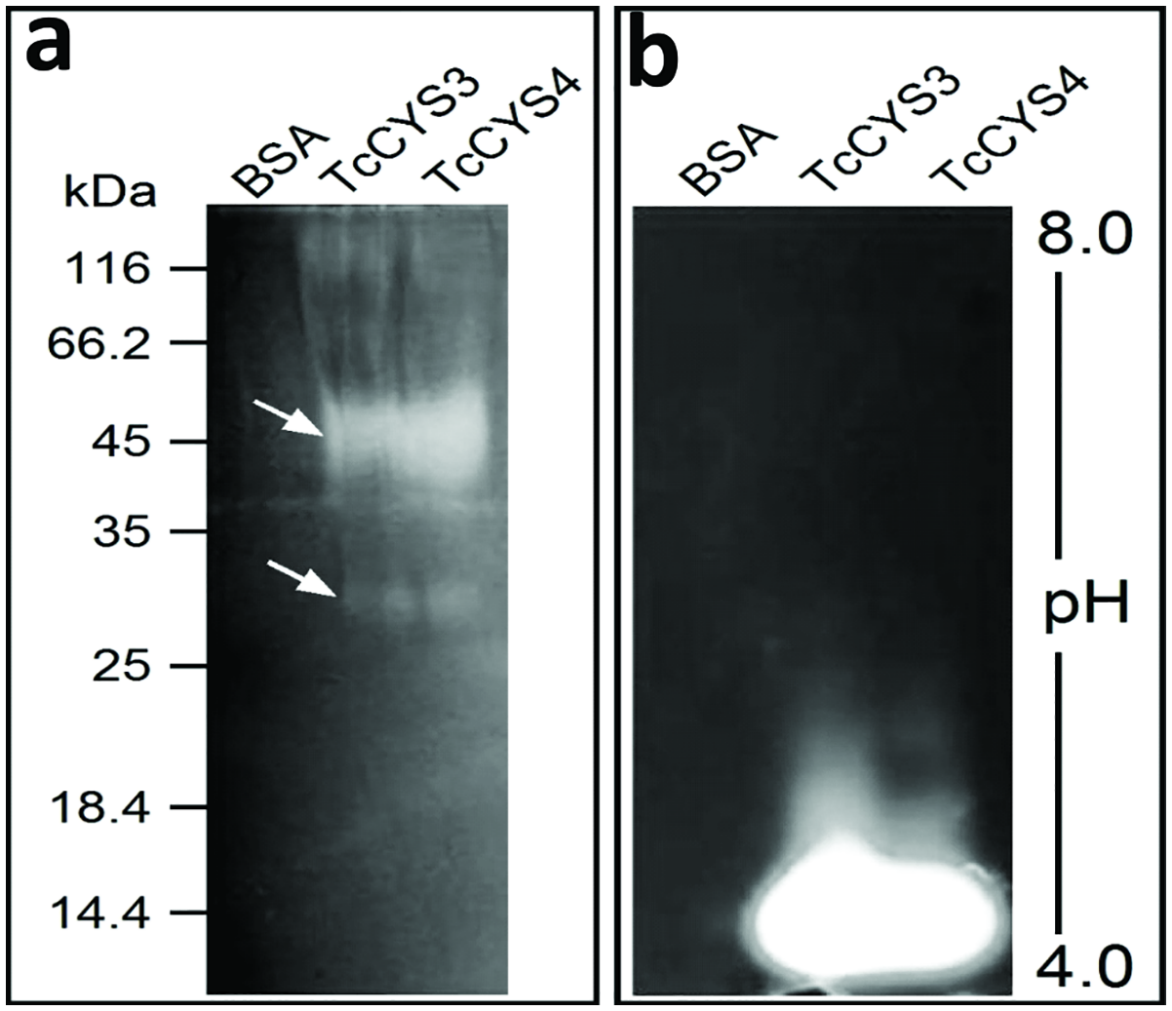
Protease activity of protein captured by TcCYS3 and TcCYS4 coupled to CNBr–Sepharose. Qualitative assay to test protease activity of samples obtained by capturing system. **a** SDS-PAGE gel showing bands of protease activity of about 30 and 45 kDa (arrows). **b** IEF gel showing clear bands of protease activity at about pH 4.0. BSA—sample captured by BSA-CNBr–Sepharose (control). TcCYS3—sample captured by recombinant TcCYS3–CNBr–Sepharose. TcCYS4—sample captured by recombinant TcCYS4–CNBr–Sepharose.

The capture systems were used to analyze the interaction between proteases from cacao and two recombinants cystatins—TcCYS3 and TcCYS4. Specific proteases in extracts of cacao were captured by cystatins coupled to CNBr-Sepharose. The captured protein showed protease activity (Fig 5). It is interesting to note that the same bands were observed on SDS-PAGE and zymogen gel electrophoresis for the two capture systems (TcCYS3 and TcCYS4), but were not detected bands to BSA-CNBr-Sepharose. Two bands were observed on gelatin/SDS-PAGE, a band less intense with molecular weight ~30 kDa and other more intense with ~ 45 kDa (Fig 5a). On zymogram gel electrophoresis were observed bands with pH 4.0 to two protease traps (Fig 5b).

### Identification of the captured proteases by mass spectrometry

The proteins captured by TcCYS4 were sequenced by mass spectrometry; 5 proteins were encountered, chlorophyll A and B binding protein (3 peptides), glyceraldehyde 3-phosphate dehydrogenase (2 peptides), rubisco (2 peptide), aldolase-like protein (1 peptide), cysteine-protease (5 peptides) (Table 1). After BLAST analysis against public databanks, the last 5 peptides showed 100% identity with TcCYSPR04 (Fig 1), which was previously identified in cacao cDNA libraries [15]. Peptides 1, 2, 3, 4 and 5 had M/Z ratios of 464.2711, 1193.2236, 1332.1821, 724.0779 and 838.3265, respectively (Table 1). The peptides blasted against ESTtik [21] and CocoaGenDB [22] identified (100% identity) only one protein encoded by CL83Contig1, which occurs in only one chromosome region (gene: Tc09_g006900 from 3.964.339 to 3.965.747 bp of chromosome 9).

**Table 1:** Peptides obtained after capture using TcCYS4 and mass spectrometry. MW: molecular weight; M/Z: mass-to-charge ratio.

Protein match	M/Z	Charge	Peak MW (Da)	Peptide MW (Da)	Start*	End*	Sequence
Chlorophyll A and B binding protein	1378.0233	3	4131.0	4130.9	54	92	(R)VLYLGPLSGDPPSYLTGEFPGDYGWDTAGLSADPETFAR(N)
	528.268	2	1054.5	1054.5	95	102	(R)ELEVIHCR(W)
	492.2465	2	982.4	982.4	124	131	(K)FGEAVWFK(A)
Glyceraldehyde 3-phosphate dehydrogenase	807.4199	2	1612.8	1612.8	84	99	(K)DSPLDVIAINDTGGVK(Q)
	627.8088	2	1253.6	1253.6	312	322	(K)TFAEEVNAAFR(D)
Rubisco	727.8732	2	1453.7	1453.7	58	69	(K)FETLSYLPDLTR(E)
	470.7253	2	939.4	939.4	104	111	(R)IPGYYDGR(C)
Aldolase-like protein	564.7853	2	1127.5	1127.5	170	179	(R)TAAYYQQGAR(F)
Cysteine protease	464.2711	2	926.5	926.5	146	154	(R)EVGIVSPVK(N)
	1193.2236	3	3576.6	3576.6	182	215	(K)GISLSEQQLVDCAGAFNNFGCNGGLPSQAFEYIK(Y)
	1332.1821	2	2662.3	2662.3	237	261	(K)FSSENVGVQVIDSVNITLGAEDELK(H)
	724.0779	3	2169.2	2169.2	262	281	(K)HAVGLVRPVSVAFEVVTSFR(F)
	838.3265	2	1674.6	1674.6	322	335	(K)NSWGEDWGDDGYFK(M)

* In relation to the target protein.

### Building and validation of the 3D model of TcCYSPR04

**Fig 6:**
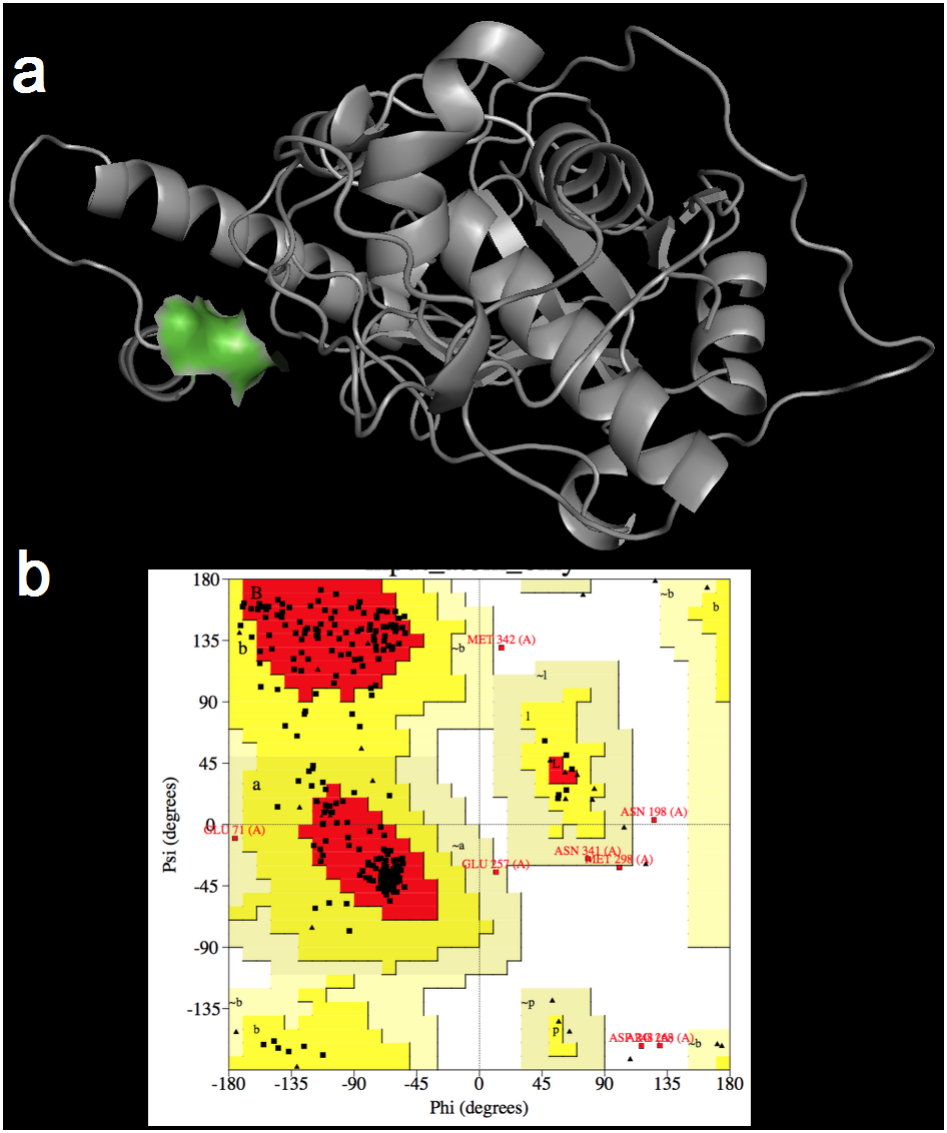
3D model of the pro-TcCYSPR04. **a** 3D model after MD simulations with detailed active site (in green) containing the C25, H165 and N185 catalytic triad plus the W187. **b** Ramachandran plot of 3D model after MD simulations with all amino acids from active site in allowed positions.

BLASTP results regarding the cysteine protease of cacao showed 1CS8 (pro-cathepsin L) from *Homo sapiens* as the best template for modeling, with 44% identity. The initial model of TcCYSPR04 has 354 residues, and presenting all previously described amino acids from catalytic site (Fig 6a). The removal of the inhibitory region has exposed the catalytic cleft of the protein, which contains the catalytic trial formed by the C25, H165 and N185 plus a W187. After refinement and MD, the 3D model of TcCYSPR04 (Fig 6b) showed a Ramachandran plot with 96.9% of residues in allowed regions.

### Building and validation of the 3D model of TcCYS4

**Fig 7:**
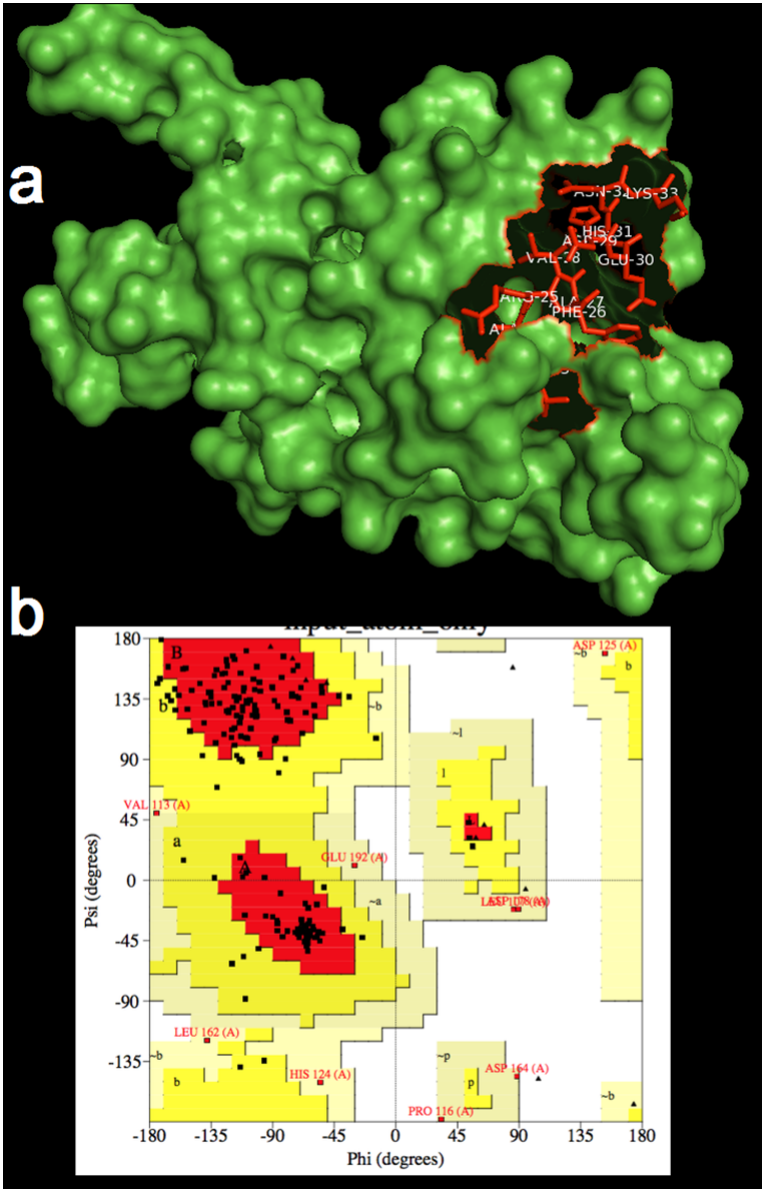
3D model of TcCYS4. **a** 3D model after MD simulations presenting the inhibitory region (in red). **b** Ramachandran plot of 3D model after MD simulations with amino acids in allowed positions, including inhibitory region.

A clone from cacao-*M*. *perniciosa* cDNA library has been identified as a putative cystatin named TcCYS4, which was fully described by Pirovani et al [14]. Briefly, the initial model of TcCYS4 showed 196 amino acids with a inhibitory region containing the QVVAG and PWMN motives as well as the conserved phytocystatin (from L23 to K33) and legumain (from S143 to L146) domains (Fig 7a). BLASTP results for the cacao cystatin (TcCYS4) showed one reliable template (4LZI) from *Colocasia esculenta* with 38.42% of identity. After refinement and MD, the 3D model of TcCYS4 showed a Ramachandran plot (Fig 7b) with 95.4% of residues in allowed regions.

### TcCYSPR04-TcCYS4 interaction model

**Fig 8:**
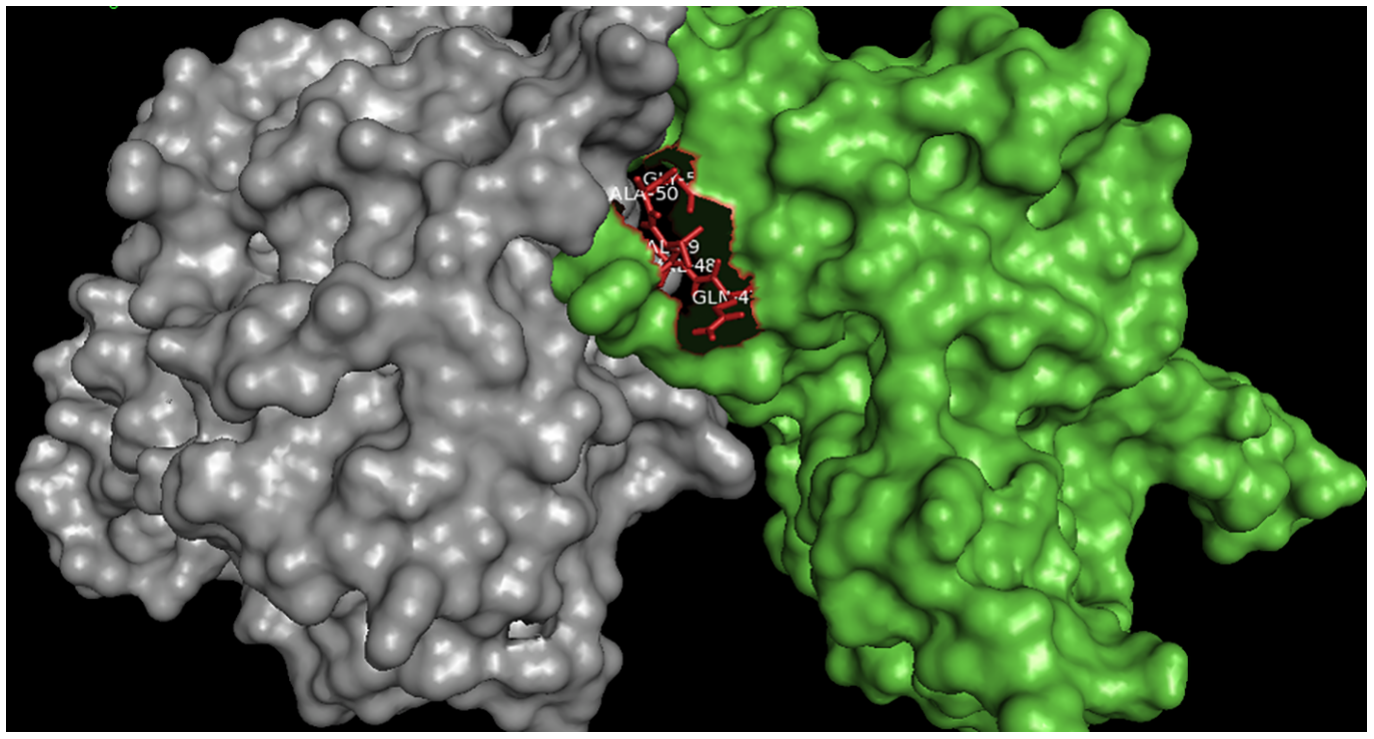
Docking between TcCYSPR04 and TcCYS4. Complex showing the catalytic and inhibitory (in red) region of TcCYSPR04 and TcCYS4, respectively.

The docking between TcCYSPR04 and TcCYS4 has resulted in various possible conformations, whereof only one—the most favorable and probable according to the selection criteria (see Material and Methods)—was chosen. This complex showed energy of -802.7 E/kT. The inhibitory QVVAG domain appears interacting with the catalytic triad of TcCYSPR04 (Fig 8). The interaction was obtained through van der Waals forces and hydrogen bonds.

## Discussion

We have identified 448 genes encoding sequences of the proteases of all classes in cacao genome [22]; cysteine-protease corresponds to 30%. The gene TcCysPR04, previously identified in the cDNA library from *T*. *cacao* and *M*. *perniciosa* interaction [15], represents one of these sequences. It has an ORF with 1,068 nucleotides in length encoded a polypeptide of 355 amino acids with a theoretical molecular mass of 39 kDa, which is in accordance with the general characteristics of the PLCPs [4].

Sequence analysis has revealed that TcCYSPR04 is a pre-pro-protein, i.e., containing a peptide signal, an inhibitory pro-region and the mature enzyme (Figs 1 and 6). The presence of peptide signal and the low pI value found (Fig 5) indicates that TcCYSPR04 is a secreted protein, as observed for many plant PLCPs [4]. Fourteen and seven putative phosphorylation and glycosylation sites, respectively, were encountered along the pro-TcCYSPR04 (Fig 1). The presence of such post-transcriptional modifications and formation of protein-protein complex may explain the differences observed between theoretical and experimental protein size observed band on SDS-PAGE gel (Figs 2–5).

The 3D model has demonstrated that the putative sites to post-transcriptional modifications were exposed on the protein surface. Post-transcriptional modifications of the pro-protein may also be involved in the pro-region cleavage and mature protein activation, as well as in the interaction of the active TcCYSPR04 with its inhibitor, TcCYS4 [45, 46]. The identification of immunoreactive bands in senescent leaves with expected size for the mature form between 27 and 36 kDa (Fig 2b) highlights the involvement of this protease in the degradation and mobilization of cellular proteins characteristic of this plant development phase [46, 47]. Senescence is controlled by gene regulation and a series of signals that start in the final phase of leaf development [48]. The regulation of senescence is explained by complex external and internal stimuli, for example, signal transduction to suppress genes responsible for photosynthesis, and activation of genes involved in the disassembly of cell structures [49].

The cysteine proteases can be synthesized as monomers and oligomerized to form dimers, trimers and tetramers during senescence, or may be complexed with cystatin [50]. The presence of bands with molecular weight higher than 50 kDa in the leaf extract of *T*. *cacao* suggests that cysteine-proteases can form protein-protein complex in addition to post-translational modification (PTM), which could increase its molecular weight. Thus, the bands with molecular weight higher than 50 kDa corroborate the predictions of glycosylation shown in Fig 1. A similar result was obtained for the electrophoretic migration of cysteine-proteases in tissues of senescent spinach, where the author suggests that the increased molecular weight of the enzyme is due to PTM [46]. This process tends to lower the protein pI and it is usually involved in activation and deactivation of enzymes. So, this inhibitor should be released in order that the enzymes undergo the PTM and/or acidification and become active by triggering senescence or resistance and program cell death (PCD) responses [14, 27].

Plants of Catongo cacao variety showed a gradual increase of the enzyme (a band of ~ 30 kDa) during leaf ontogeny in control plants. The increase of this specific band was not observed in the infected tissue, but an additional band of ~ 27 kDa appears in stage E3 (mature leaves with more than 35 days after infection) (Fig 2a). Plants may have a localized response to the infection site that prevents either the colonization of the pathogen or even a response to the mismatch, in the case of biotrophic pathogens [51]. In the interaction between *T*. *cacao* and *M*. *perniciosa*, there is evidence that the biological process of PCD could be controlled by the pathogen during infection [16]. Thus, the fungus takes advantage of the situation and suppresses the cell death [52]. The appearance of a ~ 27 kDa band in the advanced stage of infection (Fig 2a) may be related to the fungal shift from biotrophic to saprophytic, when occur the plant cell death [53].

Bands with molecular weight higher than 50 kDa were imunodetected in leaf apoplastic fluid of Catongo and TSH1188, but a band with expected molecular weight (~27 kDa) was detected only in infected sample from TSH118 (Fig 3). Such observations indicate that TcCYSPR04 is a vacuolar or apoplastic protein, as was predicted in the TargetP analyses (Fig 1). Besides this, the molecular weight of the protein detected in apoplastic fluid indicates that the protein can pass by PTM after its biosynthesis. However, the exclusive band observed in infected TSH (Fig 3) can be result of the accumulation protein unmodified, a differential response expressed by resistant genotype [19, 20]. The modifications increases the molecular weight and forming mature protease complexes (Fig 5a), as formerly proposed [50]. Furthermore, the captured protease showed activity in pI value near 4 (Fig 5b). The pI value for mature form of the protein was close to pH of the apoplast and vacuole. The apoplast is an important molecular barrier, cystatin (CC9) from maize when is transcriptionally induced is directed to apoplast where it inhibits apoplastic papain-like cysteine proteases [5].

Cysteine proteases have also responded to the treatment with MpNEP in tobacco leaves (Fig 4). MpNEP is a protein identified in the genome of *M*. *perniciosa*, in the library of the interaction between *T*. *cacao* and *M*. *perniciosa* [15]. By inducing the production of ethylene in the plant, this fungal protein induces the premature senescence process and triggers all cell death mechanisms in the plant [18, 52]. There is a series of bands in the gel immunodetected by antiserum against TcCYSPR04, suggesting, mainly 72 hours after the interaction MpNEP-plant, the participation of various isoforms of cysteine-proteases in physiological events probable related to necrosis events triggered by MpNEP. Overexpression of TcCYS4 in tobacco affected the response of plants inoculated with MpNEP2 through the action of cystatins, which inhibit cysteine proteases activated during the PCD process triggered by MpNEP2 [52]. Some of these protein-protein complexes may have been identified in our immunoblottings with anti-TcCYSPR04 and this explaining the different molecular weights detected in our samples.

The molecular battle that occurs during the development of witches' broom symptoms begins to be clarified. Our data, together with the results of Pirovani et al [14], which analyzed the accumulation of cystatin in healthy and infected tissues, have indicated that balance between cysteine-protease and cystatin in *T*. *cacao* might be a determining factor in the development of witches' broom symptoms as suggested by van der Hoorn [3] for different pathosystems. Recently, it was shown that variations in pH and temperature can affect the oligomerization of TcCYS4 modifying the inhibitory activity this cystatin against PLPCs engaged in PCD, and affect symptoms of witches’ broom disease of cocoa, caused by the fungus *M*. *perniciosa* [54]. Cystatin levels also decrease in mature tissues, while the main difference concerns the protein levels between infected and uninfected tissues during the transition from green broom to dry broom, which corresponds to the transition from biotrophic to necrotrophic mycelium of *M*. *perniciosa* [16].

We have also reported the capture of an active cysteine-protease using two recombinant cystatins (TcCYS3 and TcCYS4), both from cacao (Fig 5). It is noteworthy that this system allowed capture of the active enzyme after their interaction (Fig 5), unlike what was published to another capture system [55]. The captured proteins were sequenced and the peptides obtained showed 100% of identity to TcCYSPR04 with 48% coverage of its sequence (105 out of 218 amino acids; Fig 1). The alignment of the peptides in databanks has allowed the identification of only one cDNA and one region of the genome. Considering 134 cysteine-protease in the cacao genome, the capture of only one protease has indicated that it is probably the most abundant enzyme found in leaves or the one with the highest affinity to TcCYS4 (or both). *TcCYSPR04* was located on the chromosome 9. Interestingly, on this chromosome are located the major QTL (quantitative traci loci) of witches’ broom [56, 57] and most of the defense genes [22].

The 3D models of pro-TcCYSPR04 and TcCYSPR04 obtained by comparison between the 1CS8 and 8PCH templates, respectively, revealed that the pro-region covered the catalytic cleft of the catalytic region (Fig 6). The pro-region contained the consensus sequence SNPIR, which is known to be involved in the protein targeting to the vacuole, as observed for aleurain [58]. The pro-region also contained the consensus sequence ERFNIN present in the second α-helix and known to function as an inhibitory region [59]. These observations reinforce the assumption that the pro-region acts as an inhibitor of the catalytic region, avoiding an unduly activation of the mature protein during its transport to the apoplasm [60]. Another highly conserved motif, ANFD (AXNXFXD), was encountered in one of the loops of the pro-region, between the second and the third α-helices (Fig 1). In other cysteine-proteases, this motif, which may appear as GNFD, was essential for the correct processing of the protease precursor [61].

The replacement of Asp by Asn, Tyr, Met, Val or Glu resulted in non-functional papain [62]. This indicated the essential role of Asp in protease processing. Likewise, the mutation in the conserved GNFD motif of cathepsin L1 of *Fasciola hepatica* has reduced the folding function of the pro-enzyme [63]. Here, we verified that the pro-region cleavage has influenced the mature region conformation; the amino acids W163, EEA223-225, R281, E312, V315 and V354 were involved in different secondary structures before and after TcCYSPR04 pro-region cleavage (e.g. EEA223-225 was involved in a α-helix in the pro-TcCYSPR04 and in a loop in the TcCYSPR04), suggesting that the pro-region was effectively involved in the mature protein (Fig 6) folding [64–66]. The cleavage site of the pro-region of pro-TcCYSPR04 was predicted by Pfam analysis, by aligning and overlapping the 3D structures obtained from 8PCH and 1CS8 (Fig 6a) and by multiple alignment with other cysteine-proteases from plant, animal and microorganisms. The cleavage site corresponded to the LP138-139 (Figs 1 and 6a). The proteolytic activity of all cysteine-proteases arises from the presence of the catalytic Cys and His residues in the mature enzyme’s active center, in TcCYSPR04, C25 and H165 were encountered in the catalytic site (Fig 6a). The catalytic site also contained N185, which, along with C25 and H165 formed the catalytic trial encountered in other cysteine-proteases [3], and W187 (Fig 6a). The latter seemed to participate in the interaction between TcCYSPR04 and TcCYS4 (Fig 8).

The TcCYS4 model obtained by comparison with the 3IMA template showed one α-helix and 4 β-strands forming an antiparallel β-sheet (Fig 6). The conserved QVVAG and PW regions are known to constitute the inhibitory site of the cystatin [14,38]; also, these regions are located on the third and fifth loops of the β-sheet (Fig 6) and were observed to interact with the catalytic site (C, H, N triad plus W) of the cysteine-protease (Fig 7). Likewise, the docking of TcCYSPR04 and TcCYS4 has showed electrostatic complementarities of the surface contact (Fig 8). The same was observed for papain and cystatin S from saliva [9].

Both 3D models of TcCYSPR04 and TcCYS4 were validated using the PROCHECK and ANOLEA programs. The Ramachandran plots obtained for TcCYSPR04 and TcCYS4 before refinement showed that 91.8 and 81.9% of residues in energetically most favorable regions, respectively. After refinement, the percentage of residues in the energetically most favorable regions has decreased (81% and 75% for TcCYSPR04 and TcCYS4, respectively), but still remained high. In compensation, after refinement, the validation using ANOLEA showed a better score, indicating that the 3D models obtained were very similar to the structure of the biological active molecule [67–70]. The accuracy of the results revealed that the models had appropriate stereochemical and thermodynamic values.

## Conclusions

Physical interaction between the cysteine-protease (TcCYSPR04) and the cystatin (TcCYS4) from cacao was demonstrated *in vitro* by capture and mass spectrometry (for TcCYSPR04 identification). The balance between cystatin and cysteine protease is part of the cellular responses and both are related to the plant defense response to attack of the *M*. *perniciosa*. Furthermore, cysteine-protease may be involved in the senescence process and isoforms of TcCYSPR04 immunodetection in tissues during the phase change of the disease suggest that this protein may be associated with the PCD process that occurs in this phase. Our results can corroborate with development of the biotechnological strategy aiming disease control, as well as to improve the understanding mechanisms of the interaction. The homology modeling was obtained for both proteins, where molecular docking showed that the physical-chemical parameters estimated favors the interaction between the cacao enzyme and its inhibitor. The models developed in this study may be used for improving cacao resistance by de novo design methods.
